# Perioperative outcome, long-term mortality and time trends in elderly patients undergoing low-, intermediate- or major non-cardiac surgery

**DOI:** 10.1007/s40520-024-02717-7

**Published:** 2024-03-10

**Authors:** E. K. M. Tjeertes, T. F. W. Simoncelli, A. J. M. van den Enden, F. U. S. Mattace-Raso, R. J. Stolker, S. E. Hoeks

**Affiliations:** 1 Department of Anesthesiology, Erasmus MC University Medical Center, PO BOX 2040, 3000 CA Rotterdam, The Netherlands; 2 Division of Geriatric Medicine, Department of Internal Medicine, Erasmus MC University Medical Center, Rotterdam, The Netherlands; 3 Department of Anesthesiology, Franciscus Gasthuis & Vlietland, Rotterdam, The Netherlands

**Keywords:** Elderly patients, Surgery, Mortality, Surgical risk, Discharge destination, Length of stay

## Abstract

**Background:**

Decision-making whether older patients benefit from surgery can be a difficult task. This report investigates characteristics and outcomes of a large cohort of inpatients, aged 80 years and over, undergoing non-cardiac surgery.

**Methods:**

This observational study was performed at a tertiary university medical centre in the Netherlands. Patients of 80 years or older undergoing elective or urgent surgery from January 2004 to June 2017 were included. Outcomes were length of stay, discharge destination, 30-day and long-term mortality. Patients were divided into low-, intermediate and high-risk surgery subgroups. Univariable and multivariable logistic regression were used to evaluate the association of risk factors and outcomes. Secondary outcomes were time trends, assessed with Mantel–Haenszel chi-square test.

**Results:**

Data of 8251 patients, undergoing 19,027 surgical interventions were collected from the patients’ medical record. 7032 primary procedures were suitable for analyses. Median LOS was 3 days in the low-risk group, compared to six in the intermediate- and ten in the high-risk group. Median LOS of the total cohort decreased from 5.8 days (IQR 1.9–14.5) in 2004–2007 to 4.6 days (IQR 1.9–9.0) in 2016–2017. Three quarters of patients were discharged to their home. Postoperative 30-day mortality in the low-risk group was 2.3%. In the overall population 30-day mortality was high and constant during the study period (6.7%, ranging from 4.2 to 8.4%).

**Conclusion:**

Patients should not be withheld surgery solely based on their age. However, even for low-risk surgery, the mortality rate of more than 2% is substantial. Deciding whether older patients benefit from surgery should be based on the understanding of individual risks, patients’ wishes and a patient-centred plan.

**Supplementary Information:**

The online version contains supplementary material available at 10.1007/s40520-024-02717-7.

## Introduction

In the Netherlands, life expectancy has been rising continuously [1, 2] and the average 80-year old has a life expectancy of more than 7 years [3, 4]. This trend is also reflected in the surgical population, where the care for older persons (although often challenging) has become quite common [5]. Old age is related with a decline in physiological reserve [6, 7] and most of these patients will present themselves with more risk factors than their younger counterparts [8]. Although advantages in prehabilitation, operative techniques and perioperative management seem to improve outcome and quality of life in octogenarians, postoperative adverse events remain more common in elderly patients. Therefore, care for these patients warrants an age-appropriate comprehensive perioperative plan.

There is limited information on surgical outcomes in patients of 80 years or more [9]. Identification of health deficits associated with increased age can guide clinicians in deciding whether a patient benefits from surgical treatment.

The primary objective of this study is to investigate characteristics and outcomes of a large cohort of inpatients aged 80 years and older, undergoing non-cardiac surgery. Our secondary objective is to evaluate time trends from 2004 until 2017.

## Material and methods

### Study design

This retrospective observational study primarily is a descriptive report, investigating characteristics and outcomes of a large cohort of inpatients aged 80 years and older, undergoing non-cardiac surgery. The research is performed at a tertiary university medical centre in the Netherlands. The Medical Ethical Committee (METC) of the Erasmus University Medical Centre granted a formal statement and approved the non-interventional character of this study on September 25th, 2018. Patients were not subjected to acts, neither was any mode of behavior imposed, otherwise than as part of their regular treatment. Therefore, according to Dutch law, written informed consent for a patient to be enrolled in this study was not required [10, 11]. The study was conducted in compliance with the Helsinki declaration.

### Patient selection

Data were obtained from all consecutive patients undergoing elective or urgent (including emergency) surgical interventions from January 2004 to June 2017 in the Erasmus MC, the Netherlands. Patients of 80 years or older undergoing surgery within the mentioned study period were included. Exclusion criteria were outpatient, or short-stay procedures (i.e., hospital stay shorter than 1 day) and cardiac surgery. Data on surgical procedures were extracted from the electronic patient registration system by procedure codes. Surgical interventions frequently consisted of multiple codes. Purely administrative codes, or anesthesia-related codes, such as placement of an intravenous catheter were excluded. When multiple codes were linked to one intervention, the primary code was identified for further analysis. If a patient underwent different interventions during the study period, this resulted in multiple primary interventions, each included for analysis. However, survival analysis was performed at patient level and restricted to the patient’s first procedure.

### Baseline characteristics

Baseline characteristics included age, sex, height and bodyweight. The body mass index (BMI) was calculated using height and bodyweight: kg/m^2^ as recommended by the World Health Organization [12]. The American Society of Anesthesiologists (ASA) classification was extracted. Furthermore, type of surgery, dates of surgery, hospitalization and discharge, as well as discharge location were extracted from the electronic patient files. Surgical procedures were categorized according to ESC/ESA Guidelines into 29 surgery types and subsequently divided into low-, intermediate- and high-risk procedures (Table 1) [13]. Anaesthetic technique was documented and divided into general, locoregional, or local anesthesia, sedation analgesia, or neuraxial techniques. Finally, the postoperative ward receiving the patient after the interventions was documented. Patients either went to a general ward, an intensive care, high care, medium care, or a post anesthesia care unit (PACU). In this hospital, the PACU is a ward where anaesthetists provide clinical care during the first 24 h after surgery. This may include invasive, or non-invasive ventilation, goal-directed hemodynamic management, invasive monitoring and optimal pain management.

**Table 1:** Surgical risk estimate according to type of surgery or intervention

Low-risk: < 1%	Intermediate-risk: 1–5%	High-risk: > 5%
Superficial surgery Breast Dental Endocrine: thyroid Eye Reconstructive Carotid asymptomatic (CEA or CAS) Gynaecology: minor Orthopaedic: minor (meniscectomy) Urological: minor (transurethral resection of the prostate)	Intraperitoneal: splenectomy, hiatal hernia repair, cholecystectomy Carotid symptomatic (CEA or CAS) Peripheral arterial angioplasty Endovascular aneurysm repair Head and neck surgery Neurological or orthopaedic: major (hip and spine surgery) Urological or gynaecological: major Renal transplant Intra-thoracic: non-major	Aortic and major vascular surgery Open lower limb revascularization or amputation or thromboembolectomy Duodeno-pancreatic surgery Liver resection, bile duct surgery Oesophagectomy Repair of perforated bowel Adrenal resection Total cystectomy Pneumonectomy Pulmonary or liver transplant

CAS: carotid artery stenting; CEA: carotid endarterectomy

Surgical risk estimate is a broad approximation of 30-day risk of cardiovascular death and myocardial infarction that takes into account only the specific surgical intervention, without considering the patient’s comorbidities

ESC/ESA Guidelines [11]

### Postoperative outcomes and long-term mortality

Primary outcomes were length of stay (LOS), discharge destination and 30-day and long-term mortality. Discharge destination was defined as home versus non-home. Non-home consisted of: nursing home, rehabilitation, deceased during hospital stay, other hospital, and other or unknown. Information on mortality was assessed through the institution’s medical records and long-term mortality was based on information from the national public register. Secondary outcomes were time trend analysis for these primary outcomes.

### Data analysis

Continuous variables were presented as mean ± standard deviation (SD) when normally distributed, or as median and interquartile range (IQR) when data were skewed. Categorical variables were described with frequencies and percentages. Pearson's chi-squared test and Kruskal–Wallis test were used to measure and evaluate baseline characteristics. Univariable and multivariable logistic regression were used to evaluate risk factors for discharge destination and 30-day mortality. Potential associated variables (sex, age, ASA classification, and BMI) were entered in the multivariable model. Results are reported as odds ratio’s (OR) with a 95% confidence interval. Due to missing data in ASA classification and BMI, multivariable regression was performed in a two-step approach: without ASA and BMI (aORI) and with both variables included (aORII). Long-term survival estimates were performed using Kaplan Meier analysis at the patient-level and reported as 1-, 5- and 10-year survival estimates ± standard error.

For time trend analyses, patients were divided into four 3-year periods (2004–2015) and one 2-year period (2016–2017) respectively. For absolute counts within time trends, we analysed year 2004 up to and including 2016, as only part of year 2017 was assessed due to start of a new electronic health registration system. Differences in time trends were assessed with the Mantel–Haenszel chi-square test of linear association for categorical variables. Statistical analyses were carried out using SPSS (version 24, SPSS Inc., Chicago, Illinois). Graphs were made using R software version 3.51 (The R foundation for Statistical Computing, Vienna, Austria (2018)).

## Results

The search resulted in 19,027 procedure codes representing 8251 individual patients aged 80 years or older. After exclusion of administrative, cardiac and anaesthetic procedure codes, outpatients and short stay patients; the final study population consisted of 5179 patients who underwent 7032 procedures. Of these, 1225 (23.6%) patients underwent more than one intervention during the inclusion period. The selection process is visualized in the flowchart in Supplementary Fig. 1.

Of the 7032 procedures, 3137 (44.6%) were categorized as low-risk, 3365 (47.9%) as intermediate-risk and 530 (7.5%) as high-risk (Supplementary Fig. 2). The majority of patients had an ASA classification II (47.7%) or III (45.3%). Frequency of patients with ASA classification I and II decreased with each higher risk group (*P* < 0.001) (Table 2).

**Table 2:** Baseline characteristics and outcome

	Total (*n* = 7032)	Low-risk < 1% (*n* = 3137; 44.6%)	Intermediate-risk 1–5% (*n* = 3365; 47.9%)	High-risk > 5% (*n* = 530; 7.5%)	*P*-value	Missings *n* (%)
Female n (%)	3750 (53.3)	1785 (56.9)	1785 (53.0)	180 (34.0)	< 0.001	–
Age (median(IQR))	83.0 (81.0–86.0)	83.0 (81.0–86.0)	83.0 (81.0–85.0)	82.0 (81.0–85.0)	< 0.001	–
80–84 n (%)	4665 (66.3)	1995 (63.6)	2263 (67.3)	407 (76.8)		
85–89 n (%)	1826 (26.0)	889 (28.3)	833 (24.8)	104 (19.6)		
90 + n (%)	541 (7.7)	253 (8.1)	269 (8.0)	19 (3.6)		
BMI* (median(IQR))	25.0 (22.8–27.9)	25.3 (22.9–27.9)	24.8 (22.7–28.0)	24.7 (22.7–27.3)	0.036	4128 (58.7%)
ASA* n (%)					< 0.001	3702 (52.6)
I	112 (3.4)	54 (3.6)	51 (3.3)	7 (2.4)		
II	1590 (47.7)	810 (54.3)	666 (42.9)	114 (39.9)		
III	1510 (45.3)	595 (39.9)	770 (49.6)	145 (50.7)		
IV&V	118 (3.5)	32 (2.1)	66 (4.2)	20 (7.0)		
Anesthesia* n (%)					< 0.001	326 (4.6)
General	5437 (81.1)	2138 (70.3)	2868 (90.0)	431 (90.0)		
Sedation analgesia	56 (0.8)	40 (1.3)	14 (0.4)	2 (0.4)		
Neuraxial	411 (6.1)	197 (6.3)	194 (6.1)	20 (4.2)		
Regional	131 (2.0)	94 (3.1)	32 (1.0)	5 (1.0)		
Local	647 (9.6)	548 (18.0)	78 (2.4)	21 (4.4)		
Analgesia	24 (0.4)	24 (0.8)	0	0		
Post operation n (%)					< 0.001	527 (7.5)
General ward	4509 (69.3)	2514 (85.7)	1953 (63.3)	42 (8.6)		
PACU	1090 (16.8)	287 (9.8)	619 (18.4)	184 (37.8)		
Medium/high care	390 (6.0)	87 (3.0)	160 (4.8)	143 (29.4)		
Intensive care	516 (7.9)	44 (1.5)	354 (10.5)	118 (24.2)		
Length of stay (days) (median (IQR))	5.1 (2.0–11.3)	3.0 (1.4–6.9)	6.2 (3.2–10.8)	10.3 (6.0–17.8)	< 0.001	–
Destination n (%)					< 0.001	–
Home	5246 (74.6)	2805 (89.4)	2062 (61.3)	379 (71.5)		
Non-home	1786 (25.4)	332 (10.6)	1303 (38.7)	151 (28.5)		
Rehabilitation^a^	840 (11.9)	225 (7.2)	547 (16.3)	68 (12.8)		
Deceased	370 (5.3)	45 (1.4)	262 (7.8)	63 (11.9)		
Other hospital	494 (7.0)	27 (0.9)	453 (13.5)	14 (2.6)		
Other/unknown	82(1.2)	35 (1.1)	41 (1.2)	6 (1.1)		
Mortality 30 days n (%)	469 (6.7)	72 (2.3)	336 (10.0)	61 (11.5)	< 0.001	–
Long-term survival estimate (standard error)						–
1 year	0.768 (0.007)	0.845 (0.009)	0.711 (0.010)	0.708 (0.023)		
5 years	0.445 (0.010)	0.525 (0.016)	0.390 (0.015)	0.358 (0.032)		
10 years	0.152 (0.013)	0.214 (0.024)	0.108 (0.016)	0.117 (0.039)		

BMI: body mass index; ASA: American Society of Anesthesiologists; PACU: post anesthesia care unit

*Missing data in bodyweight and height measurements resulted in 58.7% of missing BMI in the study cohort. Other missing data were of ASA classification (52.6%), type of anesthesia (4.6%), and post-operation destination (7.5%)

^a^Nursing home, Rehabilitation centre, and Psychiatric centre

Most patients undergoing low- and intermediate-risk surgery were admitted to a surgical ward postoperatively (85.7% and 63.3%, respectively). Thirty-eight percent of patients undergoing high-risk procedures were admitted to the post anesthesia care unit. LOS increased by increasing surgical risk level; 3 days in low-risk patients, six in intermediate- risk patients and 10 days in the high-risk group (*P* < 0.001).

Overall, 5246 (74.6%) patients went home after hospital discharge. The highest percentage of patients went home in the low-risk category (89.4%), compared to 61.3% in the intermediate-risk group and 71.5% in the high-risk group (*P* < 0.001). Increasing age, surgical risk and ASA classification were independent predictors of non-home discharge destination (Table 3).

**Table 3:** Univariable and multivariable logistic regression for 30-day mortality and discharge destination

	Predictors for 30-day mortality			Predictors for non-home discharge		
	Univariable (95% CI)	Multivariable (95% CI)		Univariable (95% CI)	Multivariable (95% CI)	
	OR	aOR I*	aOR II**	OR	aOR I*	aOR II**
Gender						
Male	1.4 (1.1–1.7)	1.3 (1.1–1.6)	0.9 (0.6–1.4)	0.9 (0.8–1.0)	0.9 (0.8–1.0)	0.6 (0.5–0.8)
Age	1.01 (0.99–1.04)	1.04 (0.99–1.08)	1.06 (1.01–1.12)	1.05 (1.03–1.07)	1.05 (1.03–1.07)	1.10 (1.07–1.13)
Surgical risk						
Low	1	1	1	1	1	1
Intermediate	4.7 (3.6–6.1)	4.7 (3.6–6.1)	2.9 (1.8–4.5)	5.4 (4.7–6.1)	5.5 (4.8–6.3)	4.4 (3.6–5.5)
High	5.5 (3.9–7.9)	5.3 (3.7–7.6)	1.6 (0.8–3.5)	3.4 (2.7–4.2)	3.7 (2.9–4.6)	1.7 (1.1–2.4)
ASA						
I	1		1	1		1
II	1.2 (0.4–3.8)		1.2 (0.3–4.9)	1.5 (0.9–2.5)		2.0 (1.0–4.1)
III	2.9 (0.9–9.2)		2.2 (0.5–9.2)	3.0 (1.8–5.1)		3.9 (1.9–7.9)
IV and V	13.5 (4.0–45.6)		7.9 (1.7–36.3)	10.9 (5.7–20.7)		11.0 (4.8–25.3)
BMI						
< 18.5	3.6 (1.9–6.7)		3.2 (1.6–6.2)	1.1 (0.7–1.8)		0.8 (0.4–1.4)
18.5–25	1		1	1		1
25–30	0.6 (0.4–0.9)		0.6 (0.4–1.0)	0.8 (0.6–0.9)		0.8 (0.6–1.0)
≥ 30	7.2 (0.4–1.3)		0.8 (0.4–1.4)	0.8 (0.7–1.1)		0.9 (0.7–1.2)

OR: odds ratio; aOR: adjusted odds ratio; CI: confidence interval

^*^aORI: variables included in the model: gender, age, surgical risk, and analyses were based on 7032 patients

^**^aOR II: variables included in the model: gender, age, surgical risk, ASA, BMI, and analyses were based on 2528 patients

**Fig. 1:**
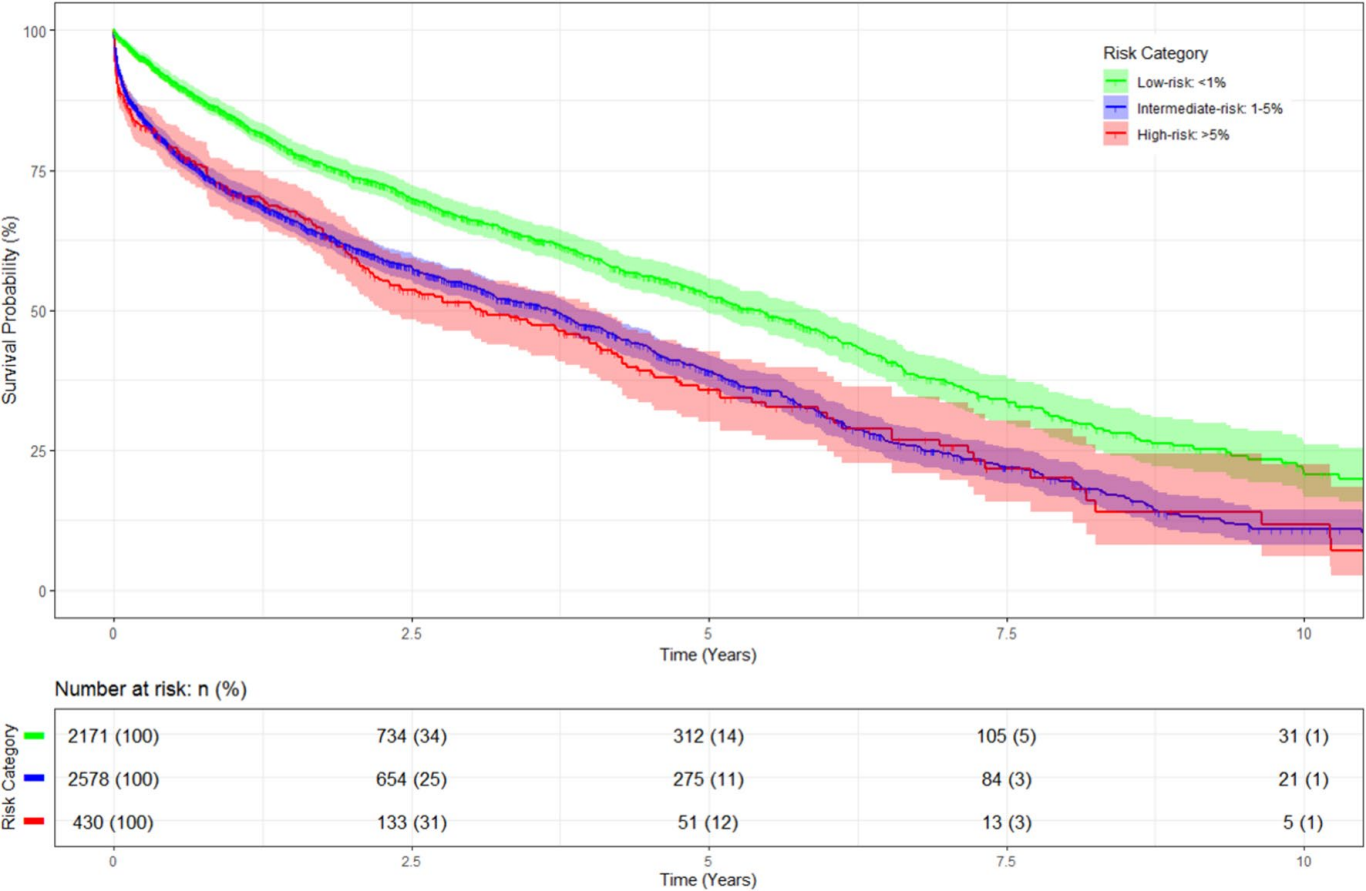
Longterm survival according to surgocal risk category

Overall 30-day mortality was 6.7% increasing from 2.3% in low-risk to 11.5% in patients undergoing high-risk procedures. Independent predictors for 30-day mortality were male sex, surgical risk, ASA classification and BMI < 18.5 kg/m^2^. A BMI 25–30 kg/m^2^ was associated with low 30-day mortality (Table 3). Median survival time of the study population (*N* = 5179, patient-level) was 4.1 years (CI 3.87–4.28) and differed across surgical risk categories, with highest survival rate in patients undergoing low-risk surgery (*P* < 0.001), Fig. 1. Survival curves for intermediate- and high-risk surgery were comparable (*P* = 0.43). The 5-year survival estimate for the low-risk group was 0.525 ± 0.016, for the intermediate-risk group 0.390 ± 0.015, and 0.358 ± 0.032 for the high-risk group, respectively. The 10-year estimates were 0.214 ± 0.024, 0.108 ± 0.016, and 0.117 ± 0.039, respectively.

**Fig. 2:**
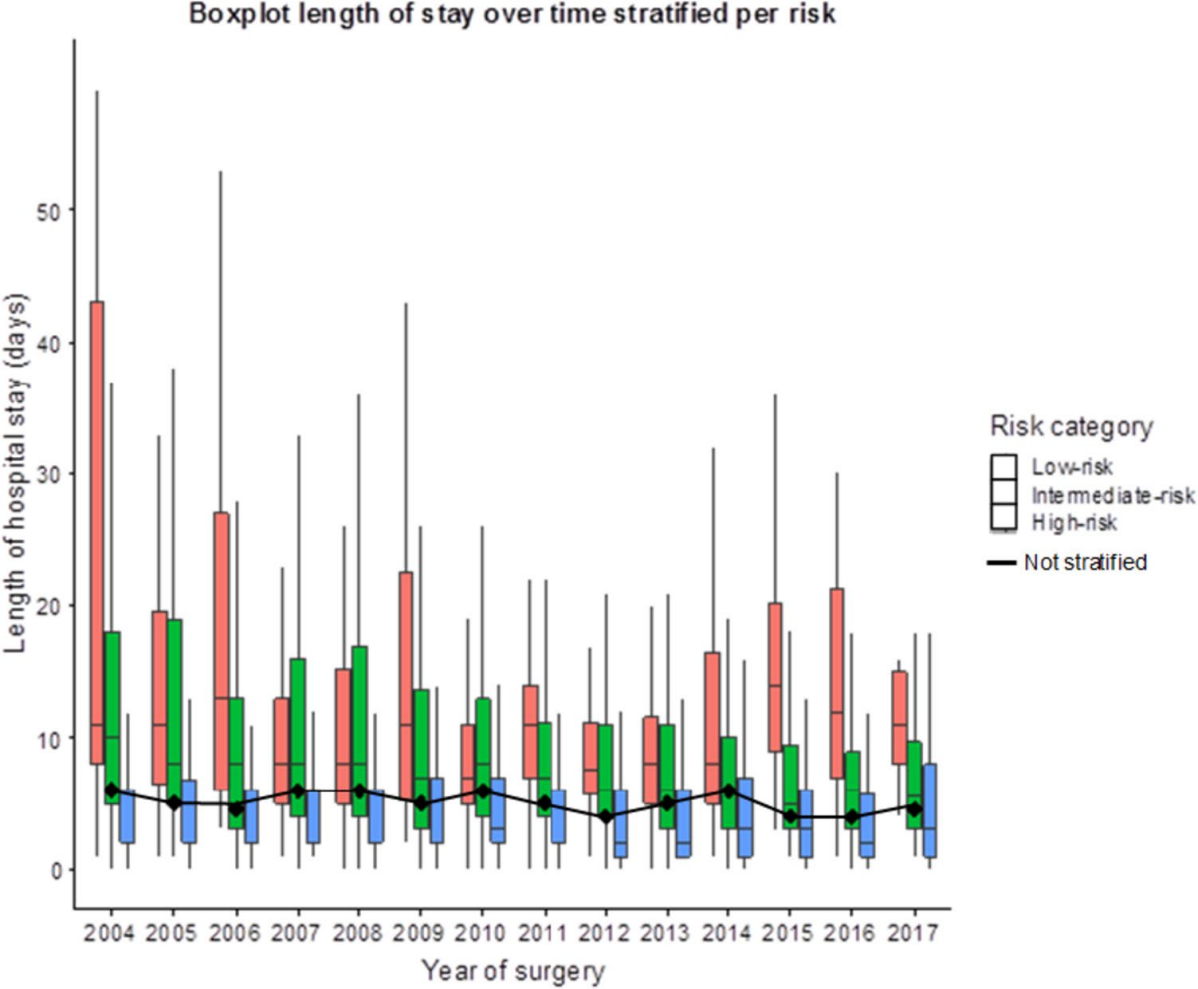
Boxplot length of stay over time, stratified per risk category

Time trends for surgical procedures showed little variation (Supplementary Fig. 3). Age distribution also showed little variation between proportions: 80–84 years varying from 65.4 to 67.5%; 85–89 years varying from 24.3 to 27.2%; and 90 years or older varying from 6.3 to 9.4% (*P* = 0.22). The LOS showed a slightly declining trend over the years. In the earliest time-group (2004–2006) the median was 5.8 days (IQR 1.9–14.5), decreased in the most recent years (2016–2017) to 4.6 days (IQR 1.9–9.0). The median LOS increased for low-risk interventions and decreased strongly in the intermediate-risk group (*P* = 0.04, *P* < 0.001, respectively) (Fig. 2).

**Fig. 3:**
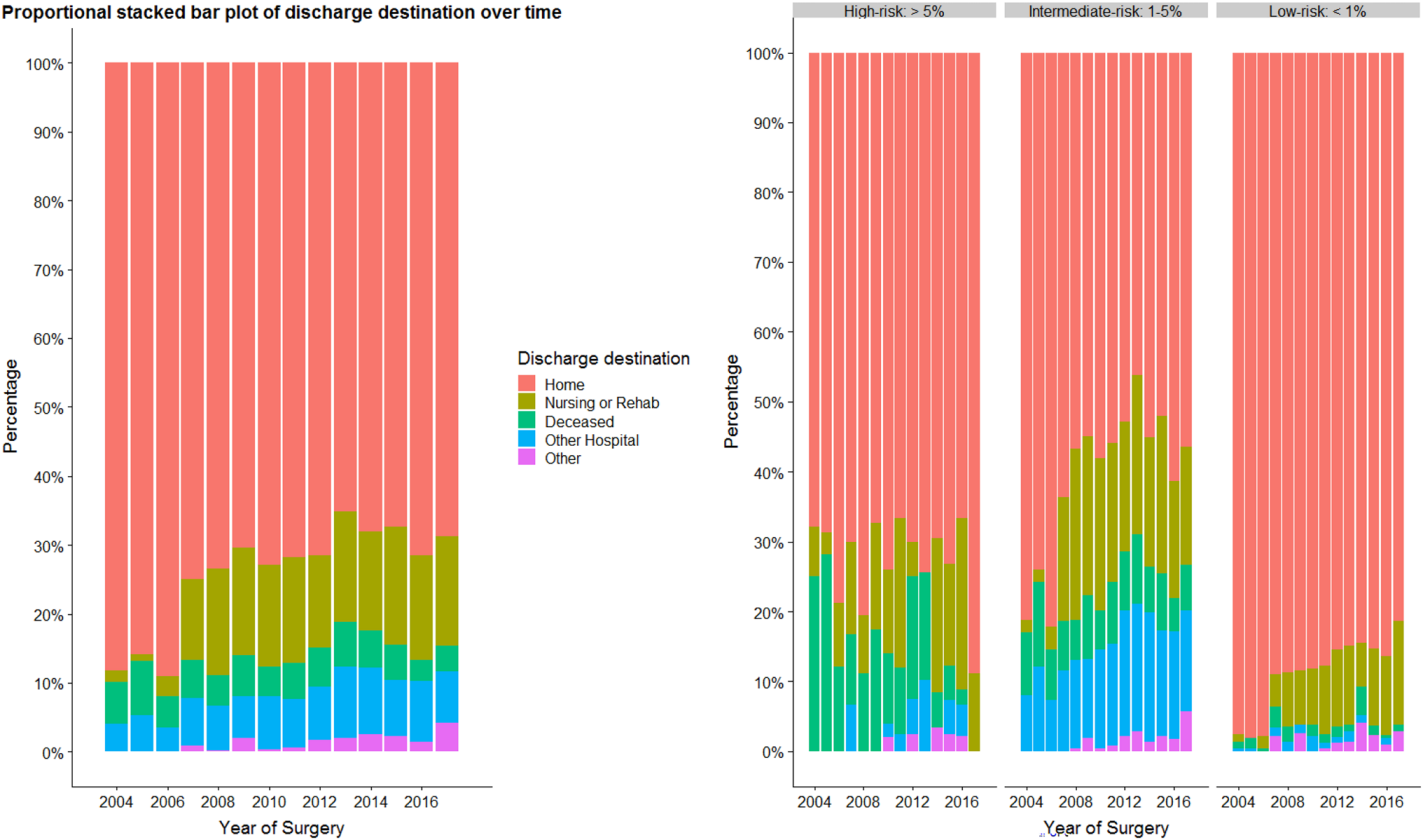
Discharge destination over time

A clear time trend regarding discharge location during the inclusion period was observed, with more patients being discharged to a specialized facility (Fig. 3). Thirty-day mortality remained rather constant over time varying from 4.2 to 8.4% (*P* = 0.36).

## Discussion

In this large observational study of 7032 procedures in 5179 elderly patients undergoing surgery, overall 30-day mortality was high (6.7%). Although patients undergoing intermediate and high-risk surgery have worse prognosis, even the 30-day mortality in the low-risk surgery group of 2.3% should not be overlooked and is higher when compared with a general population (1.8%). Particularly, since low-risk procedures are very rarely lifesaving or -prolonging, a mortality rate of more than 2% is substantial. Surgical risk and ASA-classification were associated with postoperative death and discharge to specialized facilities.

Overall LOS in this study was 5.1 days. With the evolution of surgical techniques and medical care, there is a shift towards outpatient surgical care, previously requiring hospitalization [14]. Lagergren et al. investigated outcomes after endovascular aneurysm repair in octogenarians. With comparable patient characteristics they found a similar LOS of 5.3 days [15]. Polanczyk et al. found age to be a risk factor for LOS in the hospital, noticing patients over 80 on average stayed 1 day longer [16]. Further determinants of LOS were sex, surgical risk and ASA classification [17].

After discharge, 75% of patients in this study went back home. The highest percentage of patients returning to their homes were in the low-risk category: 89.4%. In the intermediate-risk group, the percentage of patients going home was lowest. In this category more patients went to another hospital after discharge than in the other two groups (24% versus 0.9% and 2.6%). Since the hospital in this study is a tertiary academic centre, patients were referred to this hospital and sent back after surgery when considered fit enough; in the high-risk group this might not have been appropriate. Similar discharge characteristics were presented by Lagergren et al. [15]. McDonald et al. described lower rates: 62% of patients went home after hospital-stay. Since their patients mainly underwent intermediate-risk surgery, this is comparable with the 61.3% in the intermediate group of our research [18].

In the present study, we observed an in-hospital mortality of 5.3%. When looking at other studies investigating outcomes of older surgical patients, Hamel et al. found a 30-day mortality of 8.2% [9] in a population of 26,648 patients, undergoing non-cardiac surgery in a veteran hospital. Patients were predominantly classified as intermediate or high-risk, nearly all patients were men and the prevalence of ASA classification 4 was 20%, which may explain the higher mortality rate when compared to the findings of this study. Other studies including octogenarians and patients aged over 75 [19], described a 30-day mortality varying from 0.8 [18] to 8.3% [15, 19–21]. These differences can probably be attributed to surgical risk, which varied within these studies from low- to high-risk. In line with previous research, age, surgical risk and ASA classification were associated with 30-day mortality [9, 20–22]. Another association with adverse outcome found in this study was being underweight (BMI < 18.5). These patients had a threefold higher risk of postoperative mortality. These results are comparable to findings in a general non-elderly surgical population [23].

When looking at time trends, Breugom et al. described a decrease in 30-day mortality from 8.3 to 6.2% in the period 2009–2013, whereas no decline in 30-day mortality was found in our study [19]. From 2014 to 2018 the number of inpatients aged 70 years or older increased in our hospital with 14%. This upward trend was not reflected in the number of clinical surgical procedures during the study period. This can very well be explained by the exclusion of outpatients and short stay patients, which is the patient-category increasing most over the years. In the Netherlands, the total number of operations on 80-year olds and older increased from 63,866 (6.1%) in 1995 to 119,273 (8.4%) in the year 2010 [14]. In that timespan the number of inpatients remained virtually constant with an increase of 10%. The outpatients, however, undergoing mostly low-risk surgery, increased with a staggering 600%: from 8336 to 58,389 [14]. Changes in perioperative care during the study period should be mentioned. Prehabilitation, advantages in operating techniques in perioperative care have undergone tremendous developments in the last decade. The use of early warning system scores and early sense monitoring can further improve patient safety by detecting deterioration before major postoperative complications will occur.

The present study has some limitations. First, this was a single centre study with data collected in an academic tertiary referral centre. Second, only inpatients were included, leaving out many low-risk interventions. Also, due to the retrospective design of this study, we were dependent on data registered in the hospital registration system with related missing data and limited number of variables which could be automatically extracted. We used the reliable and independent ASA-score as a predictor of patients’ health. However, an important limitation is the lack of possible predictors such as comorbidities or complications, which therefor could have been underexposed. Since routine standardized assessment of frailty was not available, the presence of this comorbid condition was not taken into account. Laboratory data were recorded up to 1 year prior to the intervention, which is a broad time range in the life of an 80-year old. However, laboratory values recorded on the closest preceding date of the intervention were used, in more than 97% of cases this date was well within the year prior to surgery.

Strengths of the present study are the large number of older patients, undergoing a wide variety of surgical procedures, with different risk-profiles and long follow-up time.

McDonald et al. demonstrated that despite older age, odds can be turned with perioperative optimization of senior health leading to better outcomes [18]. Also, geriatric assessment plays an important role, covering multiple domains such as medical, mental health, functional capacity, social circumstances and environment, making it a multidisciplinary effort [18, 24, 25]. This type of care enables health care professionals to provide a patient-centred plan; optimizing preoperatively where necessary, and creating an optimal postoperative management strategy [18, 24–28]. Chow et al. described the importance to assess the patients’ capacity to provide informed consent [27]. Multiple studies show the importance for patients to maintain their functional independence [25, 27, 28]. Advance care planning should also involve patient’s short- and long-term (health) goals, and what treatment is appropriate in those cases.

Future research should probably reconsider outcome measures such as survival and length of stay as justifications for operating, since these outcomes do not provide contextual information about whether survival fulfils the patients’ goal of care, nor is it aligned with meaningful postoperative survival.

## Conclusions

Older patients present with specific health care challenges; they have physiological, pharmacological, psychological, and social attributes different than younger patients. Also, there is an emerging realization that healthcare services may need to alter their methods of care delivery to ensure age-appropriate care. Better outcomes are beneficial for patients, but can also relieve the burden of a large and growing percentage of older patients on the hospital system [29]. In accordance with recent literature, this large observational study, including patients aged 80 years and older, suggests that patients should not be withheld surgery solely based on their age [21, 30–32]. However, deciding whether an older patient benefits from surgery will often be a difficult task.

Most healthcare professionals would probably agree to perform low-risk surgery in elderly patients, even though these procedures are rarely lifesaving or -prolonging. But even for this low-risk surgery group our study shows a substantial mortality rate which should not be overlooked.

The outcome of high-risk procedures does not only depend on the pathology, but also on social factors, the patients' willingness and, most importantly, the patient's frailty status. Understanding individual potential risks, being aware of the patients’ wishes and providing patient-centred plans are key principles of good perioperative care.

### Supplementary Information

Below is the link to the electronic supplementary material.

Supplementary file 1 (DOCX 68 KB)

Supplementary file 2 (PDF 228 KB)

Supplementary file 3 (DOCX 667 KB)

## References

[1] Kontis V, Bennett JE, Mathers CD (2017). Future life expectancy in 35 industrialised countries: projections with a Bayesian model ensemble. Lancet.

[2] Christensen K, Doblhammer G, Rau R (2009). Ageing populations: the challenges ahead. Lancet.

[3] Stoeldraijer L, Van Duin C, Janssen F (2012) Bevolkingsprognose 2012–2060: Model en veronderstellingen betreffende de sterfte, Den Haag, Centraal Bureau voor de Statistiek

[4] Centraal Bureau voor de Statistiek Levensverwachting (2018) geslacht, leeftijd (per jaar en periode van vijf jaren)

[5] Statistiek CBvd Operaties in het ziekenhuis (2014) soort opname, leeftijd en geslacht, 1995–2010

[6] Saha S, Varghese S, Ahmad AA (2018). Complex valve surgery in elderly patients: increasingly necessary and surprisingly feasible. Thorac Cardiovasc Surg.

[7] Rosenthal RA, Kavic SM (2004). Assessment and management of the geriatric patient. Crit Care Med.

[8] Friedrich I, Simm A, Kotting J (2009). Cardiac surgery in the elderly patient. Dtsch Arztebl Int.

[9] Hamel MB, Henderson WG, Khuri SF (2005). Surgical outcomes for patients aged 80 and older: morbidity and mortality from major noncardiac surgery. J Am Geriatr Soc.

[10] Guideline Rijksoverheid: Niet-WMO-plichtig onderzoek en ethische toetsing (2020).

[11] Guideline KNMG: Omgaan met medische gegevens (2022).

[12] Obesity: preventing and managing the global epidemic. Report of a WHO consultation (2000) World Health Organ Tech Rep Ser 894:i-xii, 1–253 11234459

[13] Kristensen SD, Knuuti J, Saraste A (2014). 2014 ESC/ESA Guidelines on non-cardiac surgery: cardiovascular assessment and management: The Joint Task Force on non-cardiac surgery: cardiovascular assessment and management of the European Society of Cardiology (ESC) and the European Society of Anaesthesiology (ESA). Eur J Anaesthesiol.

[14] Statistiek CBvd Levensverwachting; geslacht, leeftijd (per jaar en periode van vijf jaren) (2018) Centraal bureau voor de Statistiek

[15] Lagergren E, Chihade D, Zhan H (2018). Outcomes and durability of EVAR in octogenarians. Ann Vasc Surg.

[16] Polanczyk CA, Marcantonio E, Goldman L (2001). Impact of age on perioperative complications and length of stay in patients undergoing noncardiac surgery. Ann Intern Med.

[17] Pearse RM, Moreno RP, Bauer P (2012). Mortality after surgery in Europe: a 7 day cohort study. Lancet.

[18] McDonald SR, Heflin MT, Whitson HE (2018). Association of integrated care coordination with postsurgical outcomes in high-risk older adults: the perioperative optimization of senior health (POSH) initiative. JAMA Surg.

[19] Breugom AJ, Bastiaannet E, Dekker JWT (2018). Decrease in 30-day and one-year mortality over time in patients aged ≥75 years with stage I-III colon cancer: a population-based study. Eur J Surg Oncol.

[20] Kim YW, Kim IY (2016). Factors associated with postoperative complications and 1-year mortality after surgery for colorectal cancer in octogenarians and nonagenarians. Clin Interv Aging.

[21] Turrentine FE, Wang H, Simpson VB (2006). Surgical risk factors, morbidity, and mortality in elderly patients. J Am Coll Surg.

[22] Pereira J, Pareek G, Mueller-Leonhard C (2018). The perioperative morbidity of transurethral resection of bladder tumor: implications for quality improvement. Urology.

[23] Tjeertes EK, Hoeks SE, Beks SB (2015). Obesity—a risk factor for postoperative complications in general surgery?. BMC Anesthesiol.

[24] Partridge JS, Harari D, Martin FC, Dhesi JK (2014). The impact of pre-operative comprehensive geriatric assessment on postoperative outcomes in older patients undergoing scheduled surgery: a systematic review. Anaesthesia.

[25] Vacante M, Cristaldi E, Basile F, Borzi AM, Biondi A (2019). Surgical approach and geriatric evaluation for elderly patients with colorectal cancer. Updates Surg.

[26] Partridge J, Sbai M, Dhesi J (2018). Proactive care of older people undergoing surgery. Aging Clin Exp Res.

[27] Chow WB, Rosenthal RA, Merkow RP (2012). Optimal preoperative assessment of the geriatric surgical patient: a best practices guideline from the American College of Surgeons National Surgical Quality Improvement Program and the American Geriatrics Society. J Am Coll Surg.

[28] Festen S, Kok M, Hopstaken JS (2019). How to incorporate geriatric assessment in clinical decision-making for older patients with cancer. An implementation study. J Geriatr Oncol.

[29] Etzioni DA, Liu JH, Maggard MA, Ko CY (2003). The aging population and its impact on the surgery workforce. Ann Surg.

[30] Allen J, North JB, Wysocki AP, Ware RS, Rey-Conde T (2015). Surgical care for the aged: a retrospective cross-sectional study of a national surgical mortality audit. BMJ Open.

[31] Gupta AK, Kanhere HA, Maddern GJ, Trochsler MI (2018). Liver resection in octogenarians: are the outcomes worth the risk?. ANZ J Surg.

[32] Deschka H, Machner M, Welp H, Dell'Aquila AM, Erler S, Wimmer-Greinecker G (2016). Cardiac reoperations in octogenarians: do they really benefit?. Geriatr Gerontol Int.

[33] Tjeertes EK (2021). Thesis: outliers matter.

