# Supplementary material for: Perioperative outcome, long-term mortality and time trends in elderly patients undergoing low-, intermediate- or major non-cardiac surgery
Source: Aging Clin Exp Res. 2024 Mar 10;36(1):64. doi: 10.1007/s40520-024-02717-7 (PMC10925572; doi:10.1007/s40520-024-02717-7)
Supplement: Supplementary file 1 — (DOCX 68 KB) [file 40520_2024_2717_MOESM1_ESM.docx]

**Supplementary Figure 1; Flowchart procedure codes selection**
