# Supplementary figures and images for: Perioperative outcome, long-term mortality and time trends in elderly patients undergoing low-, intermediate- or major non-cardiac surgery
Source: Aging Clin Exp Res. 2024 Mar 10;36(1):64. doi: 10.1007/s40520-024-02717-7 (PMC10925572; doi:10.1007/s40520-024-02717-7)

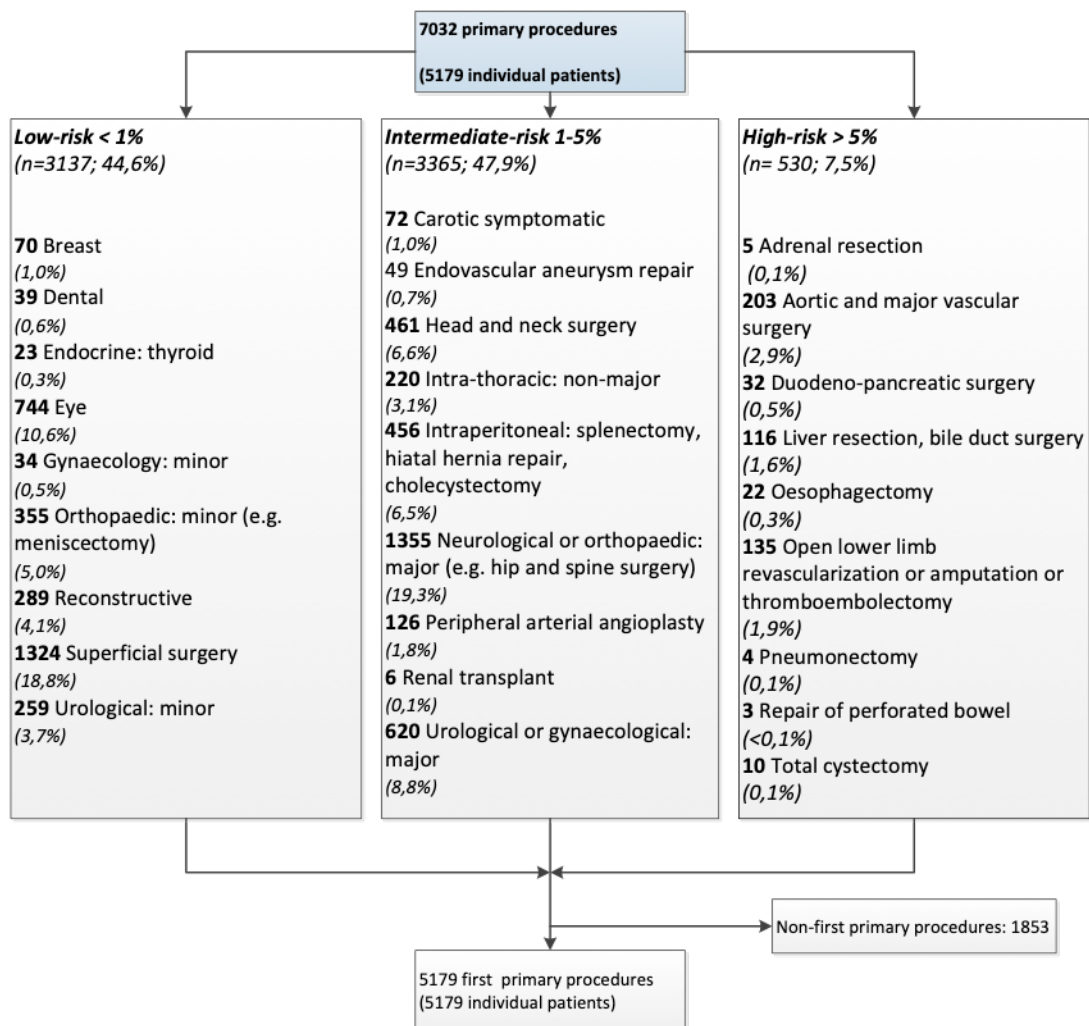

Supplement: Supplementary file 2 — (PDF 228 KB) [file 40520_2024_2717_MOESM2_ESM.pdf]

**Supplementary Figure 3; Primary procedures over time**


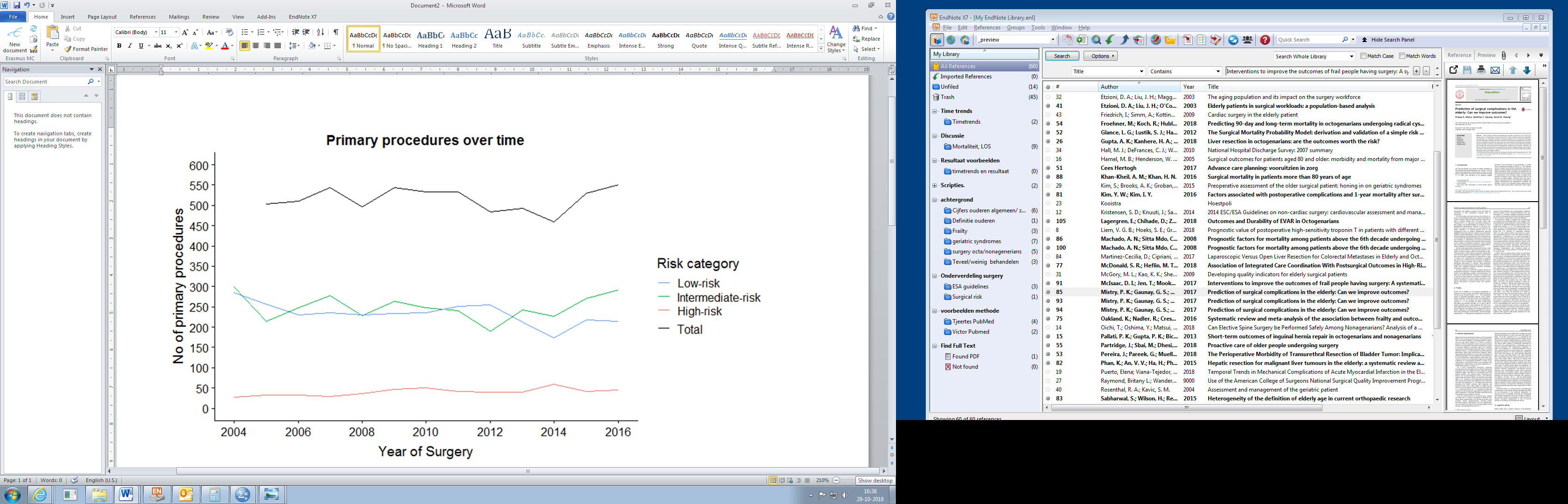

Supplement: Supplementary file 3 — (DOCX 667 KB) [file 40520_2024_2717_MOESM3_ESM.docx]
